# Rescue of Salivary Gland Function after Stem Cell Transplantation in Irradiated Glands

**DOI:** 10.1371/journal.pone.0002063

**Published:** 2008-04-30

**Authors:** Isabelle M. A. Lombaert, Jeanette F. Brunsting, Pieter K. Wierenga, Hette Faber, Monique A. Stokman, Tineke Kok, Willy H. Visser, Harm H. Kampinga, Gerald de Haan, Robert P. Coppes

**Affiliations:** 1 Section Radiation and Stress Cell Biology, Department of Cell Biology, University Medical Center Groningen, University of Groningen, Groningen, The Netherlands; 2 Section Stem Cell Biology, Department of Cell Biology, University Medical Center Groningen, University of Groningen, Groningen, The Netherlands; 3 Department of Radiation Oncology, University Medical Center Groningen, University of Groningen, Groningen, The Netherlands; 4 Department of Oral and Maxillofacial Surgery, University Medical Center Groningen, University of Groningen, Groningen, The Netherlands; University of Reading, United Kingdom

## Abstract

Head and neck cancer is the fifth most common malignancy and accounts for 3% of all new cancer cases each year. Despite relatively high survival rates, the quality of life of these patients is severely compromised because of radiation-induced impairment of salivary gland function and consequential xerostomia (dry mouth syndrome). In this study, a clinically applicable method for the restoration of radiation-impaired salivary gland function using salivary gland stem cell transplantation was developed. Salivary gland cells were isolated from murine submandibular glands and cultured *in vitro* as salispheres, which contained cells expressing the stem cell markers Sca-1, c-Kit and Musashi-1. *In vitro,* the cells differentiated into salivary gland duct cells and mucin and amylase producing acinar cells. Stem cell enrichment was performed by flow cytrometric selection using c-Kit as a marker. *In vitro,* the cells differentiated into amylase producing acinar cells. *In vivo*, intra-glandular transplantation of a small number of c-Kit^+^ cells resulted in long-term restoration of salivary gland morphology and function. Moreover, donor-derived stem cells could be isolated from primary recipients, cultured as secondary spheres and after re-transplantation ameliorate radiation damage. Our approach is the first proof for the potential use of stem cell transplantation to functionally rescue salivary gland deficiency.

## Introduction

Each year, ∼500,000 patients world-wide are treated for head and neck cancer. The majority of these patients are treated with radiotherapy either alone or in combination with other treatment modalities such as surgery and/or chemotherapy, resulting in a 5 year-survival rate of approximately 50% for non-metastatic locally advanced disease. Unfortunately, the quality of life of a large proportion of the surviving patients is severely compromised because of radiation-induced impairment of salivary gland function and consequential xerostomia (dry mouth syndrome). As a result these patients suffer from hampered speech, dental problems, difficulties with swallowing and food mastification, impaired taste and nocturnal oral discomfort [1], [2].

Salivary glands consist of several cell types: acinar cells which are responsible for water and protein secretion, myoepithelial cells surrounding the acini and ducts, and ductal cells which mainly modulate the composition of the saliva. The ductal system consists of intercalated, striated/granular convoluted tubule and excretory duct cells [3], [4]. Regeneration originates from putative stem cells residing in the ductal compartment from which complete recovery is induced within a week after ductal obstruction [5]–[7].

Irreversible hyposalivation after irradiation-induced damage is mainly caused by sterilization of these primitive glandular stem cells, which prevents replenishment of aged saliva producing cells [8]. Bone marrow-derived cells (BMCs) have been suggested as an easy accessible source for multipotent stem cells that could potentially transdifferentiate and/or repair other non-hematopoietic organs [9]–[12], including salivary glands [13]. However, the use of BMCs in solid tissue repair is surrounded by controversies and the effects are limited. Transplantation of salivary gland stem cells may be a more adequate and elegant way to therapy. Surgical removal of salivary gland tissue during lymph node dissection prior to radiotherapy could provide autologous stem cells to be transplanted post-irradiation. Although the existence of salivary gland stem cells has been well documented in a series of *in vivo* studies [14], [15], such cells have never been isolated. FACS isolated Sca-1^+^/c-Kit^+^ mouse salivary gland cells have been shown to transdifferentiate into pancreas and liver lineages [16]. Several studies have revealed that stem cells derived from tissues such as brain [17], mammary gland [18], pituitary gland [19], retina [20], skin [21], inner ear [22] and pancreas [23] can be isolated, characterized and cultured in *in vitro* floating sphere cultures. Undifferentiated cells in some of these spheres have been shown to be able to generate new tissue specific structures, e.g. mammary gland pads [24], [25]. However, the functional *in vivo* characterization of cells within these spheres has only sparsely been investigated.

In this study, we developed an *in vitro* culture system to enrich, characterize, and harvest primitive mouse and human salivary gland stem cells. After intra-glandular transplantation in mice these salivary gland cell populations containing stem cells restore saliva production to clinically relevant levels. Our approach and method can be readily adopted to explore the potential of these cells to improve saliva production in patients.

## Results

### Isolation of salivary gland stem cells

**Figure 1:**
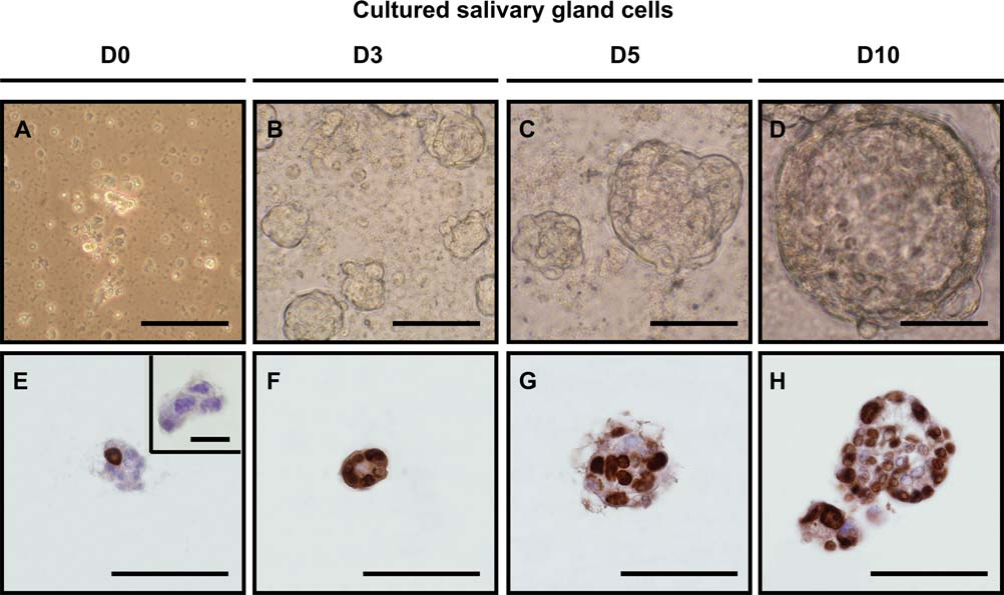
*In vitro* salisphere formation. (A) Dissociation of submandibular glands using hyaluronidase and collagenase resulted in clustered cell suspensions. 9723 ± 795 spheres were formed after 2–3 days of culturing (B), which increased in size in time (C,D). BrdU incorporation indicated that the cells in the culture were actively dividing (E–H). BrdU incorporation stained in brown, nuclei in blue. Scale bar = 50 µm. Inset shows negative control for BrdU, scale bar = 20 µm.

We developed an *in vitro* floating sphere culture system for mouse salivary gland tissue similar to methods used for other tissues [19], [26]–[32]. Small clumps of hyaluronidase and collagenase dissociated submandibular gland cells were transferred to defined DMEM/Ham's F12 medium containing EGF, FGF-2, N_2_ and insulin (Fig. 1A). Within 3 days, from the initial 2–3×10^6^ cells plated, ∼9,000 spheres per digested submandibular gland were formed (Fig. 1B). More extensive enzymatic treatment using trypsin in addition to the enzymes described resulted in a complete single cell suspension, but we were unable to culture spheres from these single cell suspensions. This suggests that initial cell–cell contact immediately after isolation is necessary for sphere formation. However, the growth of the spheres in time (Fig. 1B–D) was not due to cell aggregation but was the result of proliferation since plating of gently dissociated glands (clusters of 2–5 cells) in immobilizing semi-solid medium gave rise to sphere formation (data not shown). In addition, within spheres that were cultured up to 10 days, many cells stained positive for BrdU, indicating extensive proliferation (Fig. 1E–H). After prolonged culturing, cells detaching from spheres were predominating the culture. These detached cells were incapable of forming secondary spheres, suggesting extensive differentiation in the culture conditions used.

### Characterization of salivary gland stem cells

**Figure 2:**
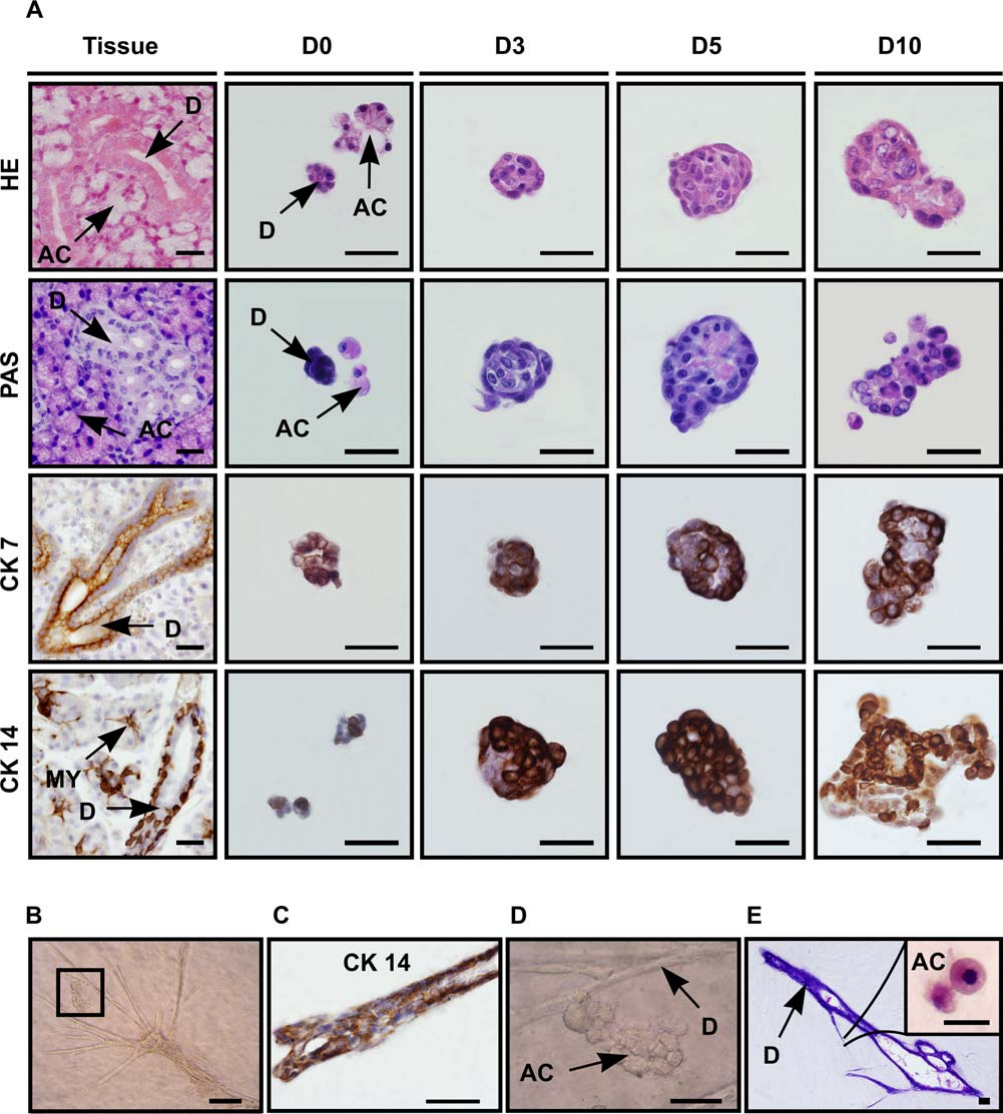
Salispheres originate from duct cells. (A) Cells from salispheres visualized by dye staining and immunohistochemistry in submandibular gland tissue (vertical lane Tissue) and after 0, 3, 5 or 10 days of culture (vertical lanes D0, D3, D5, D10, resp.). Hematoxylin-Eosin (horizontal lane HE) staining emphasized the morphology of typical submandibular gland duct (small) (D) and acinar cells (AC) recognized by their polarized nucleus. Acinar cells were present at the onset of cultivation (HE-D0), but disappeared within 3 days (HE-D3). Horizontal lane PAS: acinar presence was confirmed by PAS staining (pink) which revealed the formation of mucin/mucopolysaccharide containing cells from day 5 on (PAS-D5). Specific ductal markers CK 7 (horizontal lane CK7) and CK 14 (horizontal lane CK 14) showed the ductal origin of the sphere. When three day cultured spheres were transferred into 3D collagen formation, duct-like branches (B) appeared within the next 7 days of culture. The branches contained specific CK 14 positive duct cells (C), whereas acinar-like (D, enlargement of B) structures contained granulae and were PAS positive (E). Antibody labeling is shown in brown, nuclei in blue. Scale bar = 50 µm, inset = 20 µm. (D = ductal cell type, AC = acinar cell, MY = myoepithelial cell).

**Figure 3 pone-0002063-g003:**
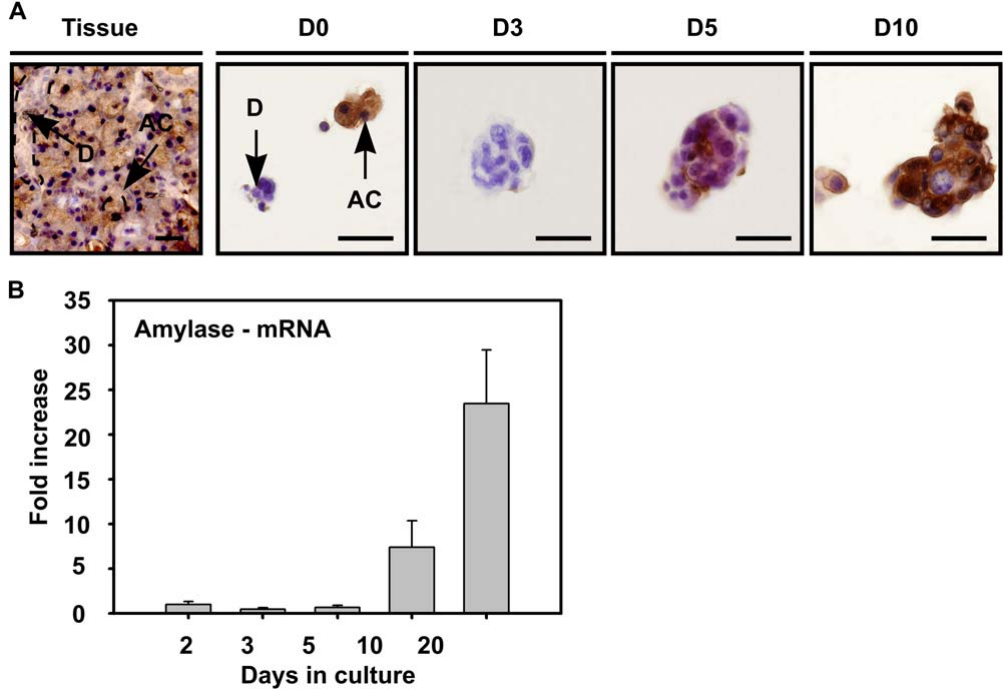
Differentiation of salisphere into acinar cells. (A) Amylase expressing cells (AC) in submandibular gland tissue (Tissue) were also present at the onset of culture (A-D0), and were visualized in the sphere at the onset of day 5 (A-D5), whereas granulae-containing spheres appeared in culture at later time-points (A-D10). Antibody labeling is shown in brown, nuclei in blue. Scale bar = 50 µm. (D = duct cells, AC = acinar cells, D0–3–5–10 represent days in culture). (B) Real time RT-PCR confirmed the enhanced expression of amylase during *in vitro* culturing and differentiation. Error bars represent SEM (*N* = 2). Amylase mRNA expression levels at 2 days of culture were normalized to one.

To characterize the origin and differentiation state of the cells in the spheres, a series of (immuno-)histochemical analyses were performed (Fig. 2). Immediately after isolation (D0) (Fig. 2A HE (Hematoxylin Eosin), PAS (Periodic Acid Schiff's base)), typical triangular shaped mucin-containing (PAS^+^) acinar cells (AC) and PAS^−^ duct cells (D) could be observed, as normally present in the tissue (Fig. 2A, Tissue). Two days later, PAS^+^ cells became undetectable in the culture, indicating selective loss of acinar cells. After 3 days, the developing spheres consisted of small cells (Fig. 2A HE: D3) with a morphology resembling glandular duct cells (Fig. 2A HE, Tissue (D)). With time, more than 90% of the spheres contained cells which had differentiated into PAS^+^ acinar like cells (Fig. 2A PAS: D5–10). At early time-points, cells in the spheres expressed the distinctive submandibular gland duct cell type markers CK 7 (Fig. 2A, CK 7) and CK 14 (Fig. 2A, CK 14), revealing the ductal origin of the cells that initiated the sphere. Strikingly, when 3 day old spheres were transferred to 3D collagen, ductal structures were formed (Fig. 2B) that expressed CK 14 (Fig. 2C). Closely associated to these ducts, morphologically distinct mucin-containing acini-like structures were formed at places distant from the original position of the sphere (Fig. 2D,E). These results indeed suggest that it is the ductal compartment of the salivary gland that contains stem cells [14], [15]. These cells were able to differentiate into acinar cells as also indicated by the increase in expression of the key enzyme amylase, analyzed either by immuno-histochemistry (Fig. 3A) and RT-PCR (Fig. 3B). Taken together, these data show that spheres are duct-derived and are capable of differentiating *in vitro* towards acinar phenotypes. From here on these spheres are referred to as salispheres.

**Figure 4:**
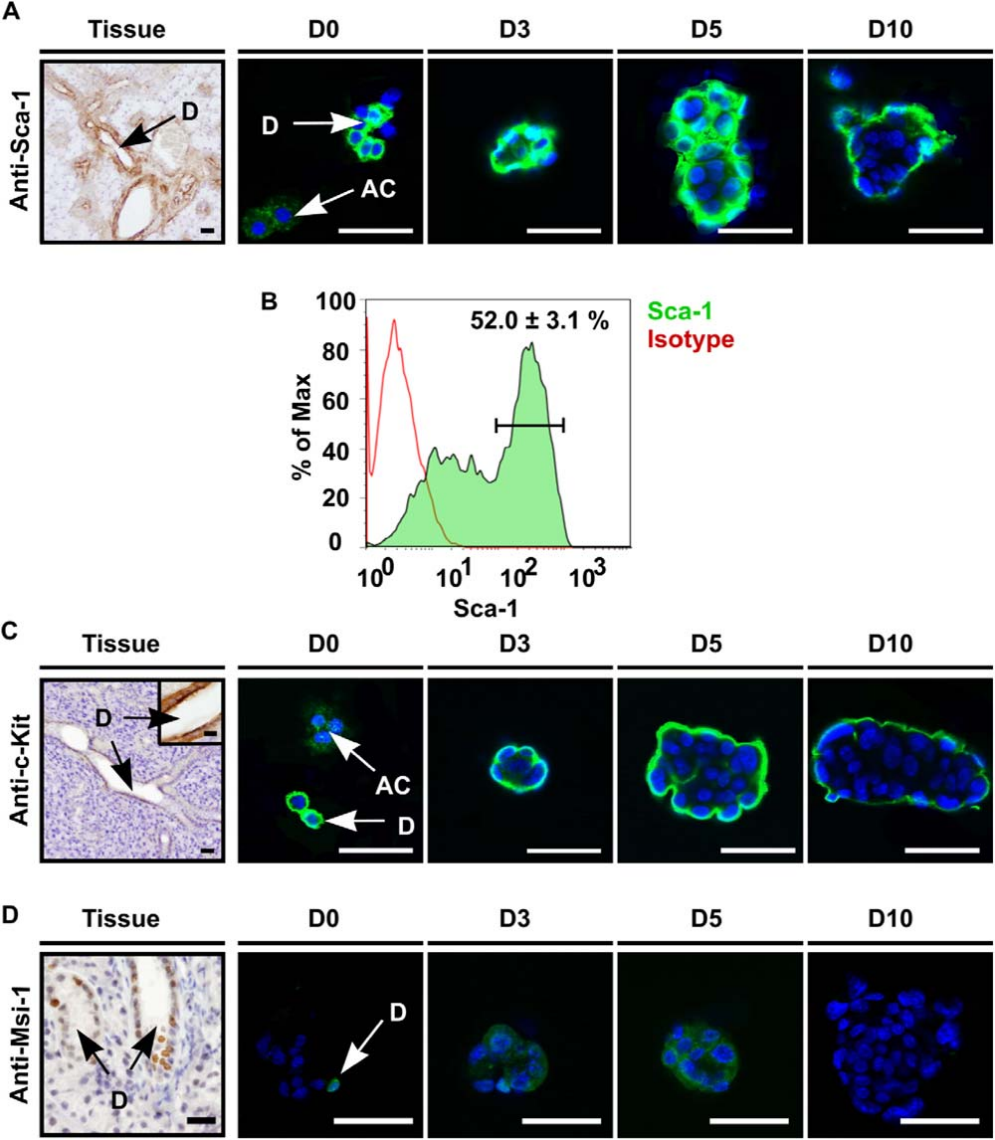
Salispheres contain stem cells. (A) Sca-1 is present in the mouse submandibular gland on endothelial cells as well as on excretory and striated duct cells (D), and was clearly present at the onset of cultivation (D0) whereas acinar cells (AC) did not express Sca-1. At day 3 and later time-points, nearly all cells at the periphery of the salispheres showed high Sca-1 expression which decreased in time (D10). (B) Approximately 52.0+/−3.1% (Mean+/−STDEV) of cells in D3 cultured spheres expressed Sca-1, as quantified by flow cytometry. (C) c-Kit is only expressed by excretory duct cells (Tissue). Salispheres showed similar c-Kit staining patterns as for Sca-1. (D) Most nuclei in excretory duct compartments and few nuclei in striated duct cells showed Musashi-1 presence (Tissue). Whereas Musashi-1 was still present in the nuclei of some cells at the onset of culturing (D0-arrow), most cells showed cytoplasmic localization which diminished in time (D3–5–10). Cells were visualized with DAPI (blue). Scale bar = 50 µm, inset = 20 µm. D = duct cells, AC = acinar cell, D0–3–5–10 represent days in culture.

Next, we explored whether salispheres indeed contain cells with stem cell characteristics. It has been shown that putative stem cells from multiple organs express Sca-1 [19], [26], [31], [32], c-Kit [30] and Musashi-1 [27]. In agreement with observations that salivary gland stem cells reside in the ductal compartment [14], [15], we observed Sca-1 expressing cells in the excretory and striated ducts but not in the acini of intact glands (Fig. 4A). At the start of cultures, Sca-1^+^ cells as well as Sca-1^−^ acinar cells were present in isolated cell clumps (D0). After 3 days almost all cells in the spheres were Sca-1^+^ with the highest expression at day 5. From day 10 on Sca-1 expression was confined to the periphery of the spheres whereas the inner acinar cell layer was Sca-1 negative. We independently confirmed Sca-1 staining patterns in the ductal compartment by evaluating GFP expression in salivary glands harvested from Sca-1/GFP knock-in mice [33] (Fig. S1). Besides ductal expression (double staining with CK 7, Fig. S1B,C), also blood vessels expressed Sca-1 as indicated by double staining with CD31 (Fig. S1D,E) or α-smooth muscle antigen (SMA, Fig. S1F,G). Sca-1 localization was confirmed to be expressed on duct cells on the periphery of cultured Sca-1 (*Ly-6A*) gland-derived spheres (Fig. S1H). Flow cytometry indicated the presence of 52.0±3.1% Sca1^+^ cells in D3 salispheres (Fig. 4B).

Expression of c-Kit was exclusively confined to the excretory ductal compartment in intact glands (Fig. 4C). Isolated cells and spheres expressed c-Kit very similar to Sca-1 (Fig. 4C: D0–10). Finally, Musashi-1 was expressed almost exclusively in the nuclei of excretory duct cells and very rarely in striated duct cells (Fig. 4D). Again, the strongest expression was observed at day 3 and 5 of culture, but disappeared at 10 days (Fig 4D: D0–10). These results strengthen the notion that primitive cells reside in the ductal compartments of the salivary gland. Furthermore, the data in figures 2–4 demonstrate that we successfully isolated and cultured salispheres that contain cells with stem cell characteristics, which were able to differentiate *in vitro* into acinar-like cells. The onset of differentiation after 5 days of culture and the gradual loss of Sca-1, c-Kit and Musashi-1 expression after 3 days of culture indicated that for subsequent functional experiments employing transplantation, cells that had been cultured for 3 days were most suitable.

### Salivary gland stem cell transplantation

**Figure 5:**
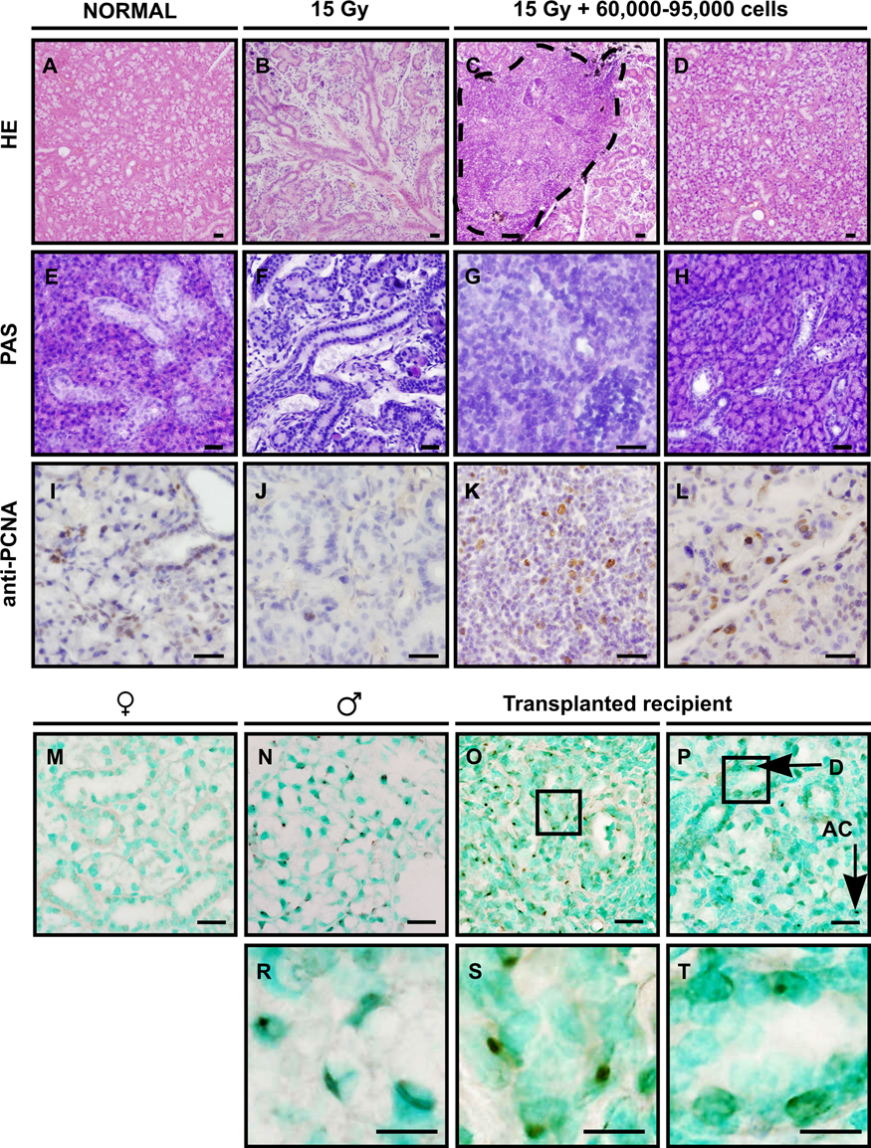
Restored morphology after injection of cultured stem cells. Single cells from day 3 salispheres were intra-glandularly injected 30 days post-irradiation and glands were analyzed 90 days post-irradiation. From left to right, normal unirradiated glands (A,E,I), irradiated non-transplanted glands (B,F,J), irradiated transplanted glands (C,G,K) at the site of injection and irradiated transplanted glands away from the injection site (D,H,L). Irradiated glands hardly contained acinar cells (B,F) as visualized by HE or PAS. In contrast, in transplanted glands, ductal cells at the site of injection (dashed line) (C,G), and acinar cells further in the tissue were readily detectable (D,H). In normal (I) and irradiated glands (J) hardly any cells stained for PCNA. In contrast, PCNA labeled proliferating cells were present at and outside the site of injection (K,L). *In situ* hybridization to detect Y chromosomes of male transplanted cells in female recipient mice. (M) No staining in female control tissue and specific staining in male control mice (N, enlargement in R). Cells at the site of injection (O, enlargement in S), as well as distant ductal cells (P, enlargement in T) and acinar cells were donor derived. Nuclei are visualized in blue or green. Scale bar = 50 µm, enlarged pictures (R,S,T) = 20 µm.

To test the *in vivo* capacity of cells obtained from cultured spheres to produce saliva, glands of female mice were irreversible damaged by 15 Gy (Gray) local irradiation. As expected, ninety days post-irradiation a pronounced loss of acinar cells was evident in irradiated non-transplanted glands when compared to unirradiated controls (Fig. 5A,B). Recipient glands were transplanted by direct intraglandular injection of dissociated cells obtained from 3 days cultured spheres, derived from male (Actbe-)GFP transgenic mice. Intra-glandular injection was used since intravenously injected cells did not home to the irradiated gland (data not shown). Prior to transplantation, approximately 60% of the injected donor cells expressed GFP, as determined by FACS analysis. Figure 5 shows the morphology of transplanted submandibular glands. From left to right, normal unirradiated glands (Fig. 5A,E,I), irradiated untreated glands (Fig. 5B,F,J), irradiated transplanted glands (Fig. 5C,G,K) at the site of injection and irradiated transplanted glands away from injection site (Fig. 5D,H,L) are shown. Ninety days after transplantation of 60–95,000 cells/gland, ductal structures were formed at the injection site (Fig. 5C, encircled). Transplanted glands appeared similar in morphology as non-irradiated glands (Fig. 5A&D) and contained a large number of acinar cells. The functionality of these cells was demonstrated using PAS staining (Fig. 5E–H). Control glands have high levels of mucin-producing cells (pink staining in Fig. 5E), which is strongly reduced after irradiation (Fig. 5F). Cells without mucins are present at the injection site (blue staining in Fig. 5G), whereas mucin-containing acinar cells colonized large areas of gland 90 days after transplantation (Fig. 5H). Whereas in normal glands (Fig. 5I) only a low number of proliferating PCNA^+^ cells was observed, in irradiated glands proliferation was almost absent (Fig. 5J). In contrast, a high number of proliferating cells was found at the injection site (Fig. 5K) and also at more distant morphologically normal-appearing tissue (Fig. 5L). This unusual high level of proliferation indicates ongoing repair of the tissue even at this late time point.

In unirradiated glands of GFP transgenic mice (Fig. S2A) GFP expression in duct cells is higher than in acinar cells. As expected, no (donor-derived) GFP expression was observed in non-transplanted irradiated glands (Fig. S2B). In reconstituted animals however, many cells expressed GFP at the site of injection 90 days post-transplantation (Fig. S2C,E). GFP expression diminished but was still detectable at more distance from the injection site (Fig. S2D,F). Anti-GFP bright field immunolabelling confirmed these results (Fig. S2G–L) and indicated that the expression of GFP was limited to ducts. This may be due to selective silencing of the actin promoter driving the ectopic expression of GFP in acinar cells. *In situ* hybridization to detect the donor Y-chromosome indeed confirmed that newly formed acinar cells were donor-derived (Fig. 5M–T). Unirradiated glands of female mice were clearly negative (Fig. 5M), whereas male glands were positive for the Y-chromosome (Fig. 5N,R). At the site of injection (Fig. 5O,S), many Y-chromosome containing male cells were detectable, as well as male acinar cells in tissue distant from this site (Fig. 5P,T). Collectively, these histological analyses revealed that radiation-damaged salivary gland tissue could be rescued by transplantation of submandibular gland-derived salisphere cells.

**Figure 6:**
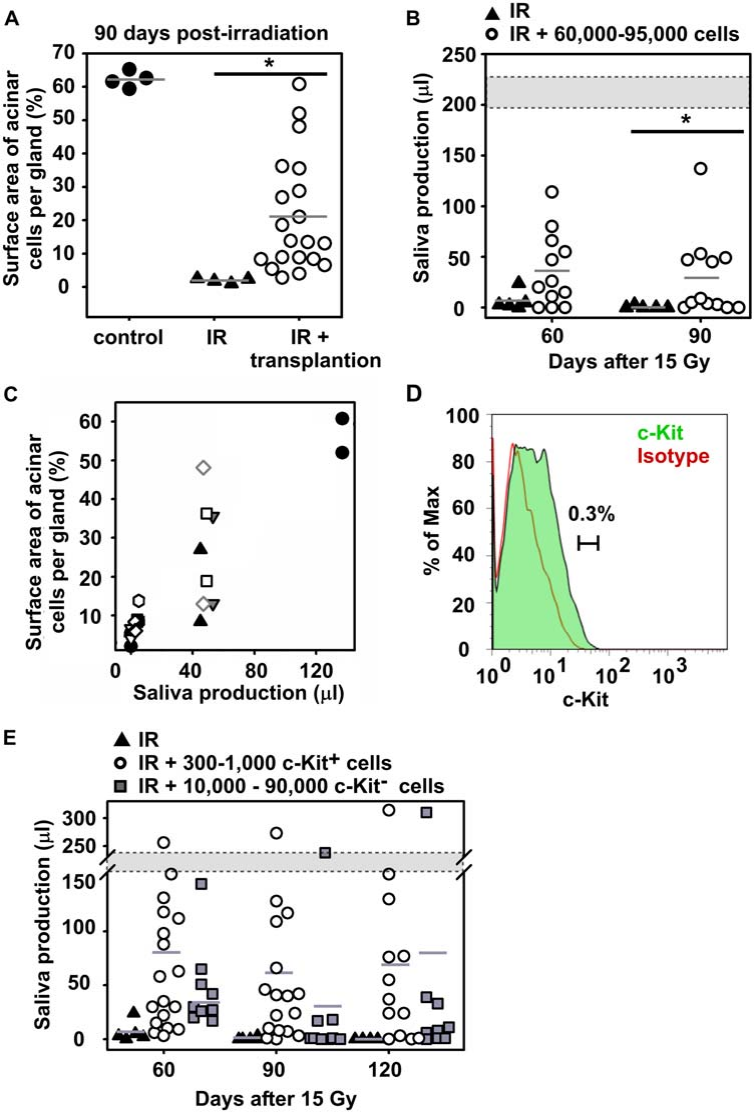
Restored organ function by transplanted progenitor/stem cells. (A) Quantifying the surface area occupied by acinar cells at 90 days post-irradiation revealed that transplanted glands contain significantly more acinar cells (○) compared to irradiated glands (▴). Saliva production measured on 90 days post-irradiation (B) was significantly higher enhanced in transplanted mice (○) compared to irradiated mice (▴). Gland function was restored in successfully transplanted mice to 23% up to 70% of normal production (gray area). (C) Saliva production correlated strongly with acinar cell restoration. Each symbol indicates a unique animal, at each time point two glands per animal were evaluated. (D) After 3 days in culture, the c-Kit brightest cells were FACS purified based on the indicated gate. (E) Injection of c-Kit^+^ cells (300 to 1,000 cells per gland) restored gland function in 9 out of 13 recipients until 120 days post-irradiation, whereas 3/9 responded when 10,000–90,000 c-Kit^−^ cells were transplanted.

Quantification of the total surface area comprising of acinar cells revealed a strong increase after salivary gland stem cell transplantation in most transplanted recipients (Fig. 6A). To assess whether these newly formed cells were fully functional, total saliva secretion after pilocarpine stimulation was measured in normal mice, and in irradiated non-transplanted and transplanted mice. Saliva production, measured 60 and 90 days post-irradiation, was significantly enhanced after cell transplantation in 42% (5/12) of the animals (Fig. 6B), compared to irradiated non-transplanted animals. Whereas unirradiated mice produced ∼220 µl saliva, successfully transplanted mice restored saliva production to 23–70% of control values. It should be noted that the total saliva flow measurement method collects saliva from parotid, sublingual and submandibular glands. However, whereas all glands have been irradiated, only the submandibular gland received a transplant. Therefore, comparison of total saliva production is likely to underestimate gland recovery after transplantation. Strikingly, a clear separation in 3 groups was observed; either very few, an intermediate number or a large number of acinar cells were found after transplant. A strong correlation (R^2^ = 0.94, *P*<0.05) between the restored acinar cell surface and quantity of saliva secreted was obtained (Fig. 6C). Saliva restoration was not achieved in all animals, which is most likely due to the technical difficulties of the injection procedure.

To further characterize salivary stem cells c-Kit^+^ cells were isolated by flow cytometry from 3 day cultured spheres. We selected the 0.3% c-Kit highest fraction of the cultured cells and compared these with the c-Kit negative population (Fig. 6D). Strikingly, intraglandular injection of only 300–1,000 c-Kit^+^ cells resulted in a pronounced improvement in saliva secretion of 9 out of 13 animals (69%) at 120 days after irradiation (Fig. 6E). In contrast, transplantation of 10,000–90,000 of c-Kit^−^ cells resulted in only 1 complete and 2 minor responders.

**Figure 7:**
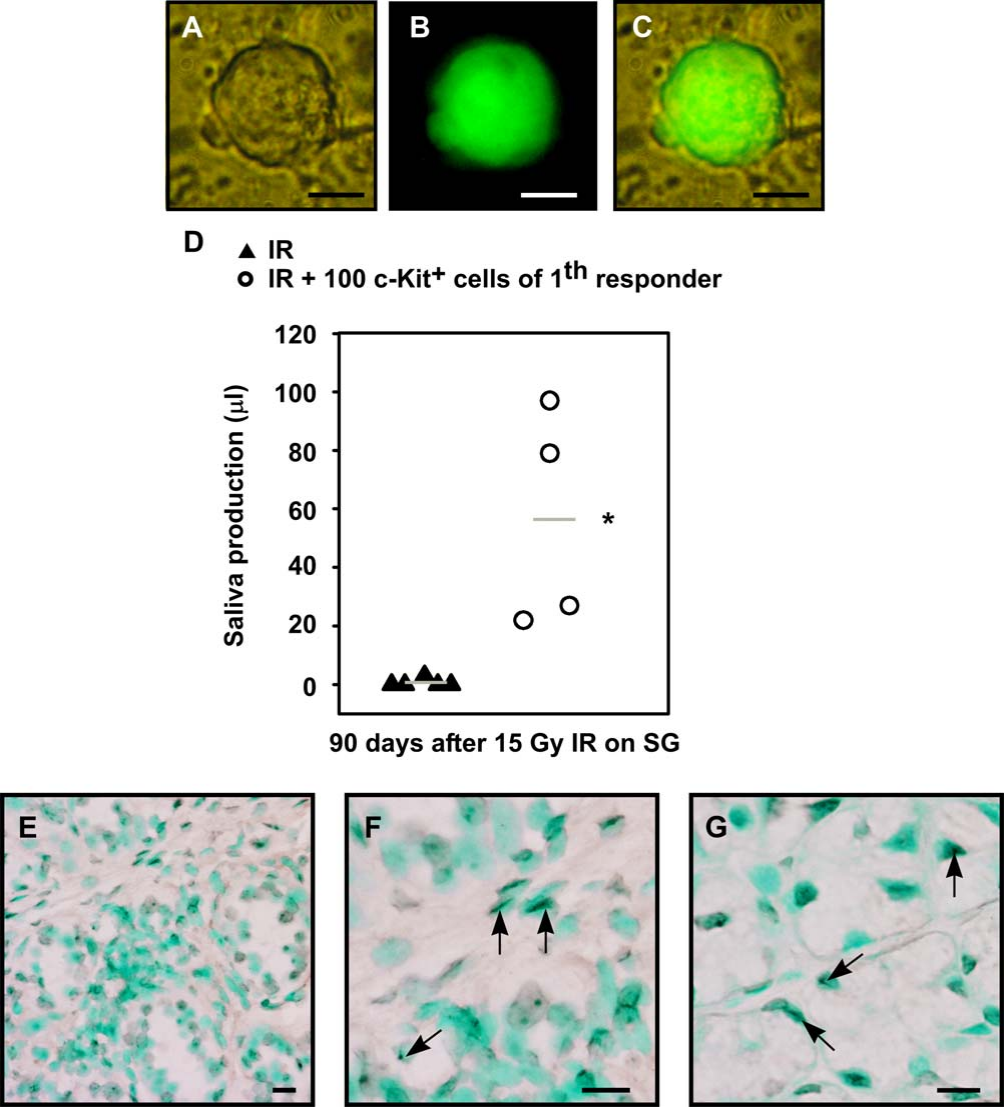
Restored organ function by transplantation of c-kit^+^ cells from secondary spheres in secondary recipients. Primary recipients were sacrificed 120 days after transplantation and secondary spheres were cultured from isolated glands. (A) Bright field image of secondary sphere, (B) GFP expression of this sphere, (C) overlay. (D) c-Kit^+^ cells were re-isolated from secondary salispheres and 100 cells were serially transplanted into irradiated gland of secondary recipients. Saliva production of secondary recipients was significantly (*P*<0.01) increased compared to irradiated control mice. (E,F,G) *In situ* hybridization to detect precence of Y chromosomes in male secondary transplanted cells in female recipient mice. Both ductal cells (F, arrows) and acinar cells (G, arrows) were Y chromosome^+^. Nuclei are visualized in green. Scale bar is 50 µm (E), or 20 µm (F,G).

Next, animals that were successfully transplanted with c-Kit^+^ cells were sacrificed 90 days post-transplantation and salivary glands were analyzed for salispheres content. Secondary spheres could be cultured from dissociated recipient glands and spheres expressing eGFP (i.e. were donor derived) could be detected (Fig. 7A,B,C). In addition, most salispheres were shown to originate from male donors, as detected by Y-chromosome specific PCR (Fig. S3). We re-purified 100 c-Kit^+^ cells obtained from these secondary spheres at day 3 and transplanted these once more. Again, an amelioration of radiation-induced gland dysfunction in secondary recipients was evident (Fig. 7D, *P*<0.01). Moreover, many Y-chromosome^+^ duct and acinar cells were observed (Fig. 7E,F,G). These results document enrichment of long-term repopulating stem cells in the c-Kit^+^ cell fraction of 3 day cultured spheres from mouse submandibular glands capable of rescuing glands from radiation damage.

To explore the potential clinical relevance of these findings, we next investigated if we could isolate a similar population from human salivary glands. Human parotid and submandibular glands were dissociated in a comparable way as mouse submandibular glands. Indeed, after 3 days of culturing human salispheres could be detected (Fig. S4A–E). Similar to mouse cells, the differentiated human acinar cells (AC) were PAS^+^ (Fig. S4G), whereas duct cells (D) and 3 days cultured spheres were not. Later in time, the cells in the spheres differentiated into PAS^+^ mucin containing cells identical to what was observed in the mouse (Fig. S4I,J). Both the human submandibular and parotid gland (Fig. S4K,L and M,N, resp.) responded similar to irradiation (Fig. S4O) as mice, with a complete depletion of acini. Furthermore, human glands also expressed c-Kit exclusively in ductal cells (Fig. S4P,Q), and a subpopulation of these c-Kit^+^ cells could be isolated from 3 day old spheres by flow cytometry (data not shown). These results indicate that a similar population of putative salivary gland stem cells can be cultured and isolated from human salivary glands.

## Discussion

Head and neck cancer is the fifth most common malignancy and accounts for 3% of all new cancer cases each year [34]. Radiotherapy alone or in combination with chemotherapy and/or surgery results in relatively high survival rates, but is also accompanied by hyposalivation, which has a severe impact on the quality of life of the patients. Here, we developed a clinically applicable method for future restoration of salivary gland function after such therapies. We show restoration of function of irreversibly damaged mouse submandibular glands after intra-glandular injection of an *in vitro* cultured c-Kit^+^ cell population containing salivary gland stem cells. Our findings raise the prospect of clinical autologous salivary gland stem cell transplantation after radiotherapy.

We were able to culture spheres from dispersed salivary glands with a similar frequency as has been described for other tissues [19]–[23], [25], [28], [29]. Cells from these spheres differentiated *in vitro* in amylase- and mucin-expressing cells. Concomitantly, the percentage of cells expressing common stem cell markers Sca-1, c-Kit and Musashi-1 decreased in time. We were unable to generate secondary spheres from the initial spheres, as has been shown for other tissues. This indicates that the self-renewal capacity of the salivary stem cells is restricted in the current culture system. However, we did not perform transplants with cells after more extended culturing periods, so it is formally possible that stem cells exist beyond three days of culturing. No salispheres were formed from single suspended cells. Apparently, cell–cell contact is necessary for salivary gland sphere formation. A similar observation has been made for intestinal stem cells [35].

Although our data indicate that the *in vitro* self-renewal capacity of the salivary stem cells seems restricted, the *in vivo* self renewal and repopulating potential of these cells is very evident. Once injected at low cell doses into glands that were irreversibly damaged by radiation, cells from spheres cultured for 3 days showed remarkable morphological and functional restoring abilities over prolonged time intervals. Repopulation of the original ductal compartment and differentiation into less primitive acinar cells was readily observed. The ability of a limited number of c-Kit^+^ cells to rescue the irradiated gland with high efficacy, the re-isolation of donor-derived cells from primary engrafted glands which can be transplanted repeatedly in successive recipients indicates that this population contains cells that meet the stringent definition of stem cells. Although the exact quantification of their *in vivo* amplification remains difficult and would rest upon several assumptions (homing efficiency, purity, functional read-out) it is evident that these cells extensively self renew *in vivo*.

To further increase the level of successful transplants several important issues remain to be explored. As indicated by the fraction of successfully transplanted mice, the c-Kit^+^ stem cell pool in the salispheres may still be heterogeneous. Further characterization of the exact cells with regenerative potential may prevent injection of a surplus of cells, which do not possess these characteristics and which in fact may negatively interfere with the engraftment.

Furthermore, improved injection techniques into irradiated atrophic salivary gland may increase the overall outcome of the stem cell transplantations. Of note, intravenously injected spheres or single cells did not home to the irradiated glands, which implies the necessity of direct gland environment. Injection into the excretory ducts of the glands may resolve this problem. We predict that it will be technically more easy to perform these transplants in rats and humans in which the glands are substantially larger.

Although we have been unable to demonstrate *in vitro* expansion of salivary gland stem cells, this may not be required for future clinical use. In analogy, hematopoietic stem cell transplantations have been practiced over the past 50 years but their *in vitro* expansion has been relatively unsuccessful, nor required [36]. Our method is unique for the treatment of irradiation-induced organ failure. Although it has been shown that salivary gland cells can be cultured and produce saliva *in vitro* [37], this has not been described in a transplantable preclinical model where the cultured cells can be harvested in adequate numbers, without contamination of other potentially damage-inflicting cells.

The presently described model appears readily transplantable to the clinic and may lead in the near future to clinically applicable use of salivary gland tissue stem cells. Our data predict that transplantation of these cells will result in amelioration of the severely reduced quality of life of surviving cancer patients. Furthermore, our approach is the first proof for the potential use of stem cell transplantation to functionally rescue solid organ deficiency.

## Materials and Methods

### Animals

Female C57BL/6 mice, 8–12 weeks old, were purchased from Harlan (The Netherlands). Enhanced GFP male C57BL/6-TgN (ActbeGFP) mice, were bred in the animal facility of the University Medical Center Groningen and used as donor mice for GFP^+^ salivary gland cells. The mice were kept under clean conventional conditions, and fed ad libitum with food pellets (RMH-B, Hope Farms B.V., Woerden, The Netherlands) and acidified tap water (pH = 2.8). Salivary glands of female Sca-1/*Ly-6* C75BL/10×DBA transgenic GFP^+^ mice were kindly provided by Prof. dr. Dzierzak of the Cell Biology and Genetic Department, Medical Center, Erasmus University, Rotterdam, the Netherlands. All experiments were approved by the Ethical Committee on animal testing of the University of Groningen.

### Isolation of salivary gland cells

Mice were euthanized via terminal anesthesia (N_2_O/O_2_/isoflurane) after blood removal (via heart punction). Submandibular glands were dissected carefully, without contamination from other tissues. Cell suspensions were prepared by mincing and enzymatically dissociation by adding collagenase type II (0.025%), hyaluronidase (0.04%) and CaCl_2_ (6.25 mM) at 37°C for 40 minutes with gentle mechanical movement. After an additional 40 minutes of fresh enzyme digestion, the tissue cell suspension was filtered with a 100 µm and 50 µm mesh using a 25G needle, and plated in non-coated 12-wells plates at 400,000 cells per well. Per gland 2.4 to 3×10^6^ cells were isolated. The culture medium consisted of DMEM/F-12 (Gibco, Invitrogen, Carlsbad, CA; 41966-029, 21765-029), penicillin, streptamycin, glutamax, EGF (20 ng/mL), FGF-2 (20 ng/mL), N_2_ supplement (1/100), insulin (10 µg/mL) and dexamethasone (1 µM). Fresh medium was added every three days. All growth factors were purchased from Sigma-Aldrich (St. Louis, MO), except for N_2_ (Gibco, Invitrogen, Carlsbad, CA). To obtain total single cell suspensions an additional treatment of 0.05% trypsin-EDTA (Gibco, Invitrogen, Carlsbad, CA; 25300) was used that released 3.6 to 4.8×10^6^ cells per gland.

The incorporation of BrdU (Sigma-Aldrich, St. Louis, MO) was determined by adding 10 µM BrdU to the culture medium for 24 h. After BrdU addition, the cells were collected and embedded in paraffin for BrdU and other immuno-labeling processes.

Rat tail collagen (2.5 mg/mL, Roche, Indianapolis, MI; 1179179) was mixed with 3 day old spheres and cultured for seven additional days until branches and acinar-like outgrowth could be visualized. CK 14 immuno-histochemistry was used as a marker for duct cells, and PAS for acinar cells.

Cytospots of undissociated spheres or single cells obtained from cultured spheres were made by spotting on glass slides through centrifugation at 400 rpm for 2 minutes.

*In vivo* visualization of GFP expression in salivary glands or *in vitro* cultured spheres was accomplished by the use of a fluorescence microscope (Olympus IMT-2, Japan) or Confocal Laser Scanning Microscopy (CSLM) (Leica TCS SP2), with or without PKH-26 cell linker visualization (Sigma-Aldrich, St. Louis, MO, P-9691).

Human normal parotid and submandibular salivary gland tissue was obtained from patients (after informed consent) with a squamous cell carcinoma of the oral cavity in whom a neck dissection procedure was performed. Care was taken to obtain only tissue from normal salivary glands. None of the patients had received any other cancer treatment before the surgical procedure. Culture conditions for the formation of human salispheres were similar to the mouse.

### Immuno-histochemistry and immuno-fluorescent analysis of spheres and salivary gland tissue

Cells were processed in paraffin following HistoGel (Richard-Allan Scientific, Kalamazoo, MI; HG-4000-012) embedding. Five-µm sections were dewaxed and labeled for the following markers: cytokeratin 7 (CK 7) (Monosan, Burlingame, CA; Mon3007), cytokeratin 14 (CK 14) (Abcam, Cambridge, MA; ab7800), BrdU (Abcam, Cambridge, MA; ab6326), Sca-1 (R&D Systems, Minneapolis, MN; AF1226), PCNA (Dako, Carpinteria, CA; M0879), c-Kit (R&D Systems, Minneapolis, MN; MAB1356), Musashi-1 (Chemicon, CA, AB5977), amylase (Sigma-Aldrich, St. Louis, MO; 8273), GFP (Chemicon, Temecula, CA; MAB3580), and GFP FITC (Abcam, Cambridge, MA; ab6662). Visualization for bright field microscopy was accomplished by adding specific secondary biotin carrying antibodies (Dako, Carpinteria, CA), an avidin-biotin-horse radish peroxidase complex (Elite ABC Kit, Vector Laboratories, Burlingame, CA) and the diaminobenzidine (DAB) chromogen. Nuclear counterstaining was performed with hematoxylin or methylene green. Control sections without primary antibodies were all negative.

Cytospots made of cultured cells or salivary gland tissues were labeled for visualization under Confocal Laser Scanning Microscopy (CSLM) (Leica TCS SP2), using avidin-FITC (Sigma-Aldrich, St. Louis, CA; A2050) and nuclear staining with 4,6-diamino-2-phenylindole (DAPI).

Cell morphology was visualized by routine histologic techniques using hematoxylin-eosin staining. Mucin and mucopolysaccharide containing acinar cells were detected by Periodic Acid Schiff's base (PAS).

### *In situ* hybridisation

The Y chromosome was stained by fluorescence *in situ* hybridization (Cambio, Cambridge, United Kingdom; 1187-YMB) according to manufacturer's instructions, but with 5 minutes of 1 mol/L sodium thiocyanate and 0.4% pepsin instead of 10 minutes. Nuclear staining was done with methylene green. Negative controls were included in the protocols.

### Irradiation and saliva collection of the mouse salivary glands

Salivary glands were locally irradiated with a single dose of 15 Gy of X-rays (Philips CMG 41 X, 200 kV, 10 mA, 5 Gy/min.). This dose is known to induce sufficient damage without compromising the general health of the animals.

At 60, 90, and 120 days post-irradiation whole saliva flow rate was determined. Animals were placed in a restraining device [38] after pilocarpine injection (2 mg/kg, s.c.). Saliva was collected for 15 minutes, and the quantity was determined gravimetrically, assuming a density of 1 g/mL for saliva.

### Flow cytometric analysis and cell purification

Cultured cells were dissociated by 0.05% trypsin-EDTA (Gibco 25300, Invitrogen) with mechanical use of 26G needles. Anti-mouse Sca-1 FITC (BD Biosciences Pharmingen-553335) and anti-mouse c-Kit FITC (BD Biosciences Pharmingen-553354) or anti-human c-Kit APC (BD Biosciences Pharmingen-550412) antibody incubation was performed at 4°C for 20 minutes, followed by a wash step in PBS/0.2% BSA. Finally, Propidium Iodide (PI, 1 µg/mL) was added to the cells before analysis using a FACS Calibur Flow Cytometer (BD) with at least 100,000 events for each measurement. Data were analyzed by FlowJo software (Tree Star, Ashland, OR). Gates for viable Sca-1^+^ or c-Kit^+^ cells were set by using isotype-controls (BD Biosciences Pharmingen) for APC, FITC and PI. Cell sorting for c-Kit^+^ cells was performed using a MoFlo flow cytometer (Dako, Carpinteria, CA), and only 0.3% of the brightest c-Kit expressing cells were selected.

### Intra-glandular injection of cultured cells

For transplantation studies, day 3 spheres were collected and dissociated by 0.05% trypsin-EDTA. Cells were suspended in an equal volume of α-MEM (Gibco, Invitrogen, Carlsbad, CA; 22561-021) containing 2% of fetal calf serum, Gibco, Invitrogen, Carlsbad, CA; 10099-141) and Indian Ink (1/200) solution to visualize the injected fluid. Both submandibular glands of irradiated mice were injected 30 days post-irradiation with 5 µL suspension containing single cells (60,000–95,000 cells per gland), undissociated spheres (4,000–7,000), sorted c-Kit^+^ (300–1,000, or 100 in case of secondary recipients), or c-Kit^−^ (10,000–90,000) cells, using a 28G needle and a Microliter Syringe (Hamilton, Reno, NV) under anesthesia.

### Quantification of acinar cells in the salivary gland

Tissue sections of glands were analyzed using bright field microscopy (Olympus CX40, Germany) under 400× magnification using 100 squares of 0.25 mm^2^ each. The percentage of surface area occupied by acinar cells was counted from two different sections (upper-middle) of each gland.

### Gene Expression Analysis by RT-PCR

Total RNA was isolated from salispheres at different time-points using the RNeasy Micro Kit (Qiagen, Hilden, Germany) according to the manufacturer's instructions. RNA concentration was measured spectophotometrically. Reverse transcription was performed on 600 ng of total RNA using oligo dT primers and M-MLV Reverse Transcriptase in a final volume of 20 µL (Invitrogen, Carlsbad, CA) for 5 minutes at 65°C, followed by one hour at 37°C. Samples were subsequently heated for 15 minutes at 70°C to terminate the reverse transcription reaction. Real-time quantitative PCR was performed on the cDNA samples using a Bio-Rad iCyler iQ Real-Time Detection System. The following primer sequences were used for *gapdh*: sense primer, 5′ ATG GCC TTC CGT GTT CCT AC 3′ and antisense primer, 5′ GCC TGC TTC ACC ACC TTC TT 3′ (accession no. NM_001001303) and for *amylase*: sense primer, 5′ GGTGCAACAATGTTGGTGTC 3′ and antisense primer, 5′ ACTGCTTTGTCCAGCTTGAG 3′ (accession no. NM_007446). Real-time PCR was conducted by amplifying the cDNA with the iQ SYBR Green Supermix (Bio-Rad, Hercules, CA). The PCR-efficiency was determined for each of the primers. Melting curve analysis of amplification products was performed at the end of the PCR reaction to confirm that a single PCR product was detected. For every PCR reaction, GAPDH was used as the internal control. Quantification of the samples was calculated from the threshold cycle (Ct) by interpolation from the standard curve. The experiments were repeated twice.

### Nested y chromosome specific PCR

Spheres were cultured from submandibular glands of transplanted recipients. As controls, spheres from submandibular gland of control male and female mice were used.

After 3 days of culture, individual spheres were selected by micromanipulation, and subjected to Y-chromosome specific PCR. Single spheres were lysed in 5 µL 400 ng/µL proteinase K/17 µM SDS. One µL of the lysate was used for nested PCR using the following primer sequences: *Y-chromosome* sense primer, 5′ AATTGACAGCATCTACGTACTGGAGC 3′ and antisense primer, 5′ TCCAGGAGCTGATAAGCATAGAGAGC 3′, second sense primer 5′ AGCTCTACAGTGATGACAGGATTTTAAACC3′, second antisense primer 5′ TGACCTCAGAGCCATCTTTCCTCTC 3′.

Nested PCR was conducted by amplifying the DNA with the Platinum Blue PCR Supermix (Invitrogen).

### Statistical analysis

The results were analyzed using a Mann-Whitney test or *t*-test. Statistical significance was defined as *P*<0.05 using SPSS. Numbers represent average±SEM, unless otherwise specified.

## Supporting Information

Figure S1 Sca-1 Expression in ductal and endothelial cell types. Expression of Sca-1 on endothelial and duct cells was confirmed using Sca-1(Ly-6A) GFP transgenic mice. (B,C) The GFP signal overlapped with the specific duct cell marker CK 7 (C enlargement of B) or CD31^+^ (D, enlargement in E), and α-smooth muscle actin myoepithelial (MY) cells (F, enlargement in G) remained negative for GFP expression. (H) Culture of Sca-1(Ly-6A) GFP salivary gland cells revealed GFP expression in duct cells whereas polarized acinar cells were negative (D0-inset). After 3 days of culture, only cells at the periphery of the sphere showed high Sca-1 GFP expression (D3). Cells were visualized with DAPI (blue, A–G) or PKH-26 (H, red). Scale bar  =  50 µm, inset  =  20 µm. D  =  ductal cell type, MY  =  myoepithelial cell, BV  =  blood vessel, D0 and D3 represent days in culture.
(6.27 MB DOC)

Figure S2 Visualization of gfp expression by fluorescence and bright field microscopy. (A) GFP was not expressed in all cells of glands from GFP transgenic donor mice, and was absent in recipient irradiated glands from normal mice prior to transplantation (B). GFP^+^ transplanted cells were present in the injected area (C, enlargement in E), and GFP was also detected in duct compartments in the surrounding area (D, enlargement in F). Fluorescent GFP data were confirmed using anti-GFP antibody (G–L) for light microscopy. Scale bar  =  50 µm, inset  =  20 µm. Nuclei are stained in blue.
(8.34 MB DOC)

Figure S3 Genotyping secondary spheres. Agarose gel showing nested-PCR products of spheres cultured from transplanted recipients contain cells with donor Y-chromosome marker. PCR on the X-chromosome and the GAPDH gene was used to verify DNA loading.
(1.50 MB DOC)

Figure S4 Human salivary glands contain c-kit^+^ cells and can yield spheres. (A–E) Dissociated human submandibular and parotid (F) glands form spheres. Initially these cells lacked mucins (PAS^−^) (G,H), but they differentiate in time into mucin expressing cells (I,J, PAS^+^). Normal human submandibular glands (K,L) contain mucous PAS^+^ and serous cells, whereas normal human parotid gland contain serous acini (M,N). (O) Irradiated human submandibular gland lose acinar cells very similar to the mouse. Both human submandibular (P) and parotid (Q) glands express c-Kit exclusively in duct cells. D  =  ductal cell type, AC  =  acinar cell, D0–3–5–10 represent days in culture. Scale bar  =  50 µm, inset  =  20 µm.
(9.12 MB DOC)
