# Supplementary figures and images for: Rescue of Salivary Gland Function after Stem Cell Transplantation in Irradiated Glands
Source: PLoS One. 2008 Apr 30;3(4):e2063. doi: 10.1371/journal.pone.0002063 (PMC2329592; doi:10.1371/journal.pone.0002063)

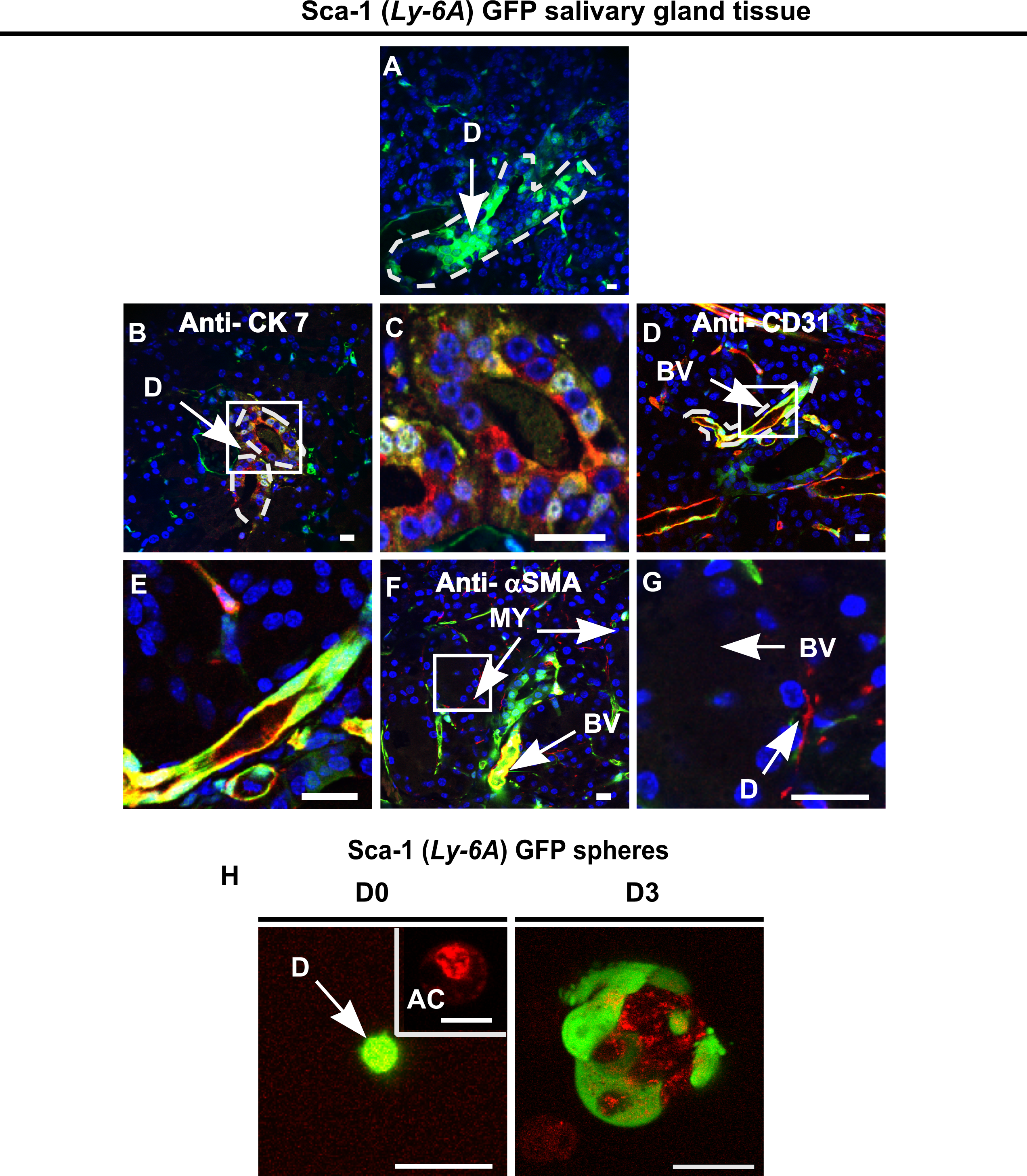

Supplement: Figure S1 — Sca-1 Expression in ductal and endothelial cell types. Expression of Sca-1 on endothelial and duct cells was confirmed using Sca-1(Ly-6A) GFP transgenic mice. (B,C) The GFP signal overlapped with the specific duct cell marker CK 7 (C enlargement of B) or CD31+ (D, enlargement in E), and α-smooth muscle actin myoepithelial (MY) cells (F, enlargement in G) remained negative for GFP expression. (H) Culture of Sca-1(Ly-6A) GFP salivary gland cells revealed GFP expression in duct cells whereas polarized acinar cells were negative (D0-inset). After 3 days of culture, only cells at the periphery of the sphere showed high Sca-1 GFP expression (D3). Cells were visualized with DAPI (blue, A–G) or PKH-26 (H, red). Scale bar = 50 µm, inset = 20 µm. D = ductal cell type, MY = myoepithelial cell, BV = blood vessel, D0 and D3 represent days in culture. (6.27 MB DOC) [file pone.0002063.s001.tif]

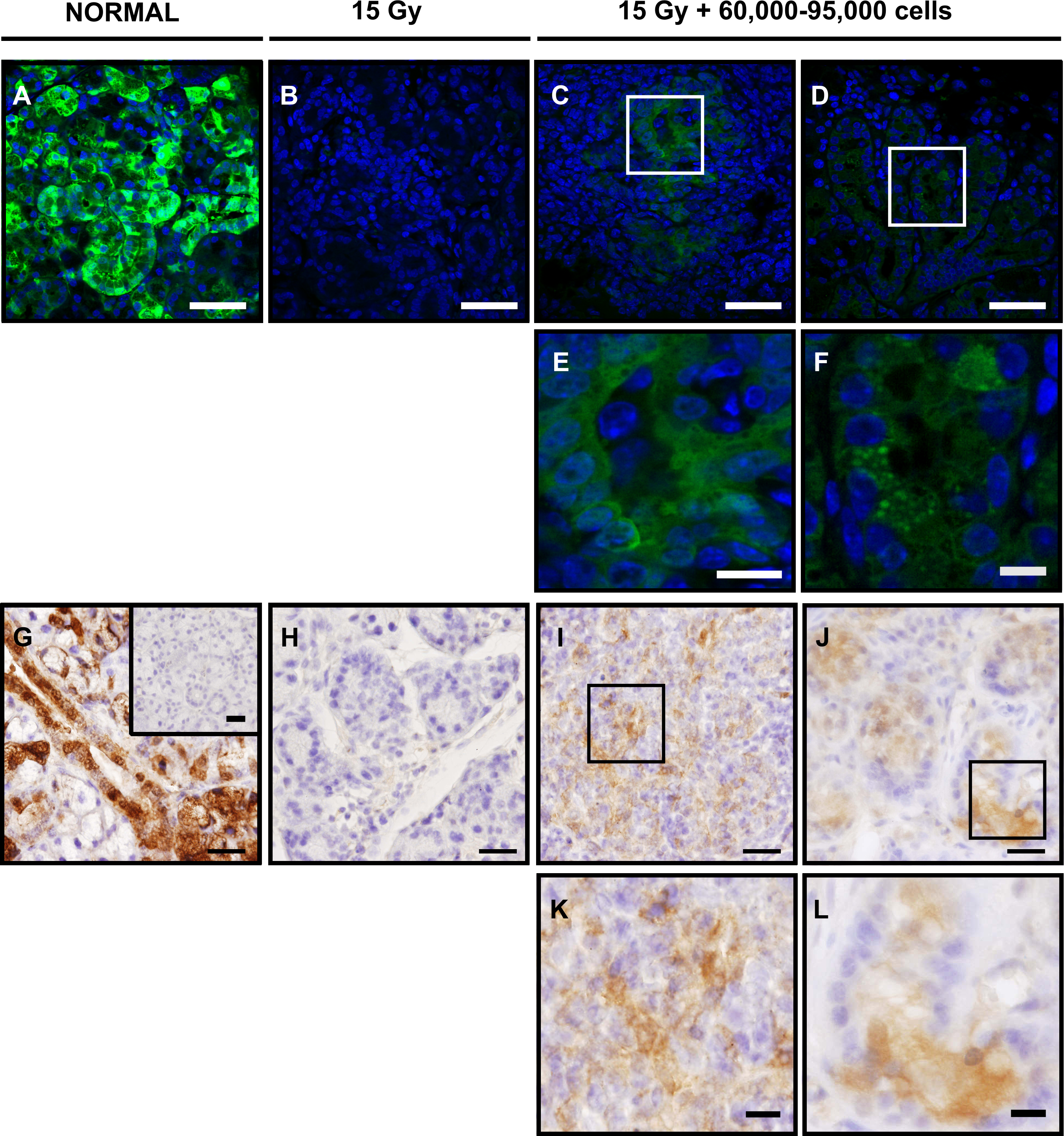

Supplement: Figure S2 — Visualization of gfp expression by fluorescence and bright field microscopy. (A) GFP was not expressed in all cells of glands from GFP transgenic donor mice, and was absent in recipient irradiated glands from normal mice prior to transplantation (B). GFP+ transplanted cells were present in the injected area (C, enlargement in E), and GFP was also detected in duct compartments in the surrounding area (D, enlargement in F). Fluorescent GFP data were confirmed using anti-GFP antibody (G–L) for light microscopy. Scale bar = 50 µm, inset = 20 µm. Nuclei are stained in blue. (8.34 MB DOC) [file pone.0002063.s002.tif]

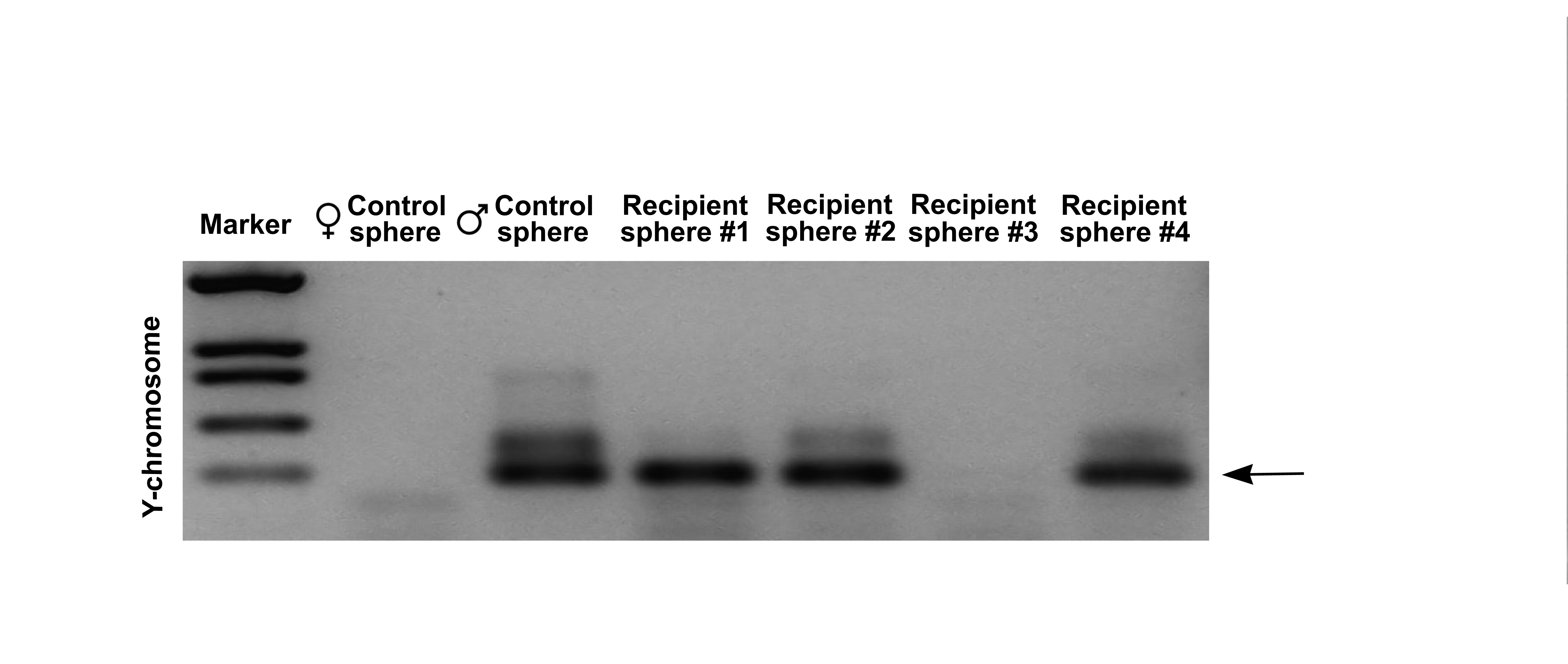

Supplement: Figure S3 — Genotyping secondary spheres. Agarose gel showing nested-PCR products of spheres cultured from transplanted recipients contain cells with donor Y-chromosome marker. PCR on the X-chromosome and the GAPDH gene was used to verify DNA loading. (1.50 MB DOC) [file pone.0002063.s003.tif]

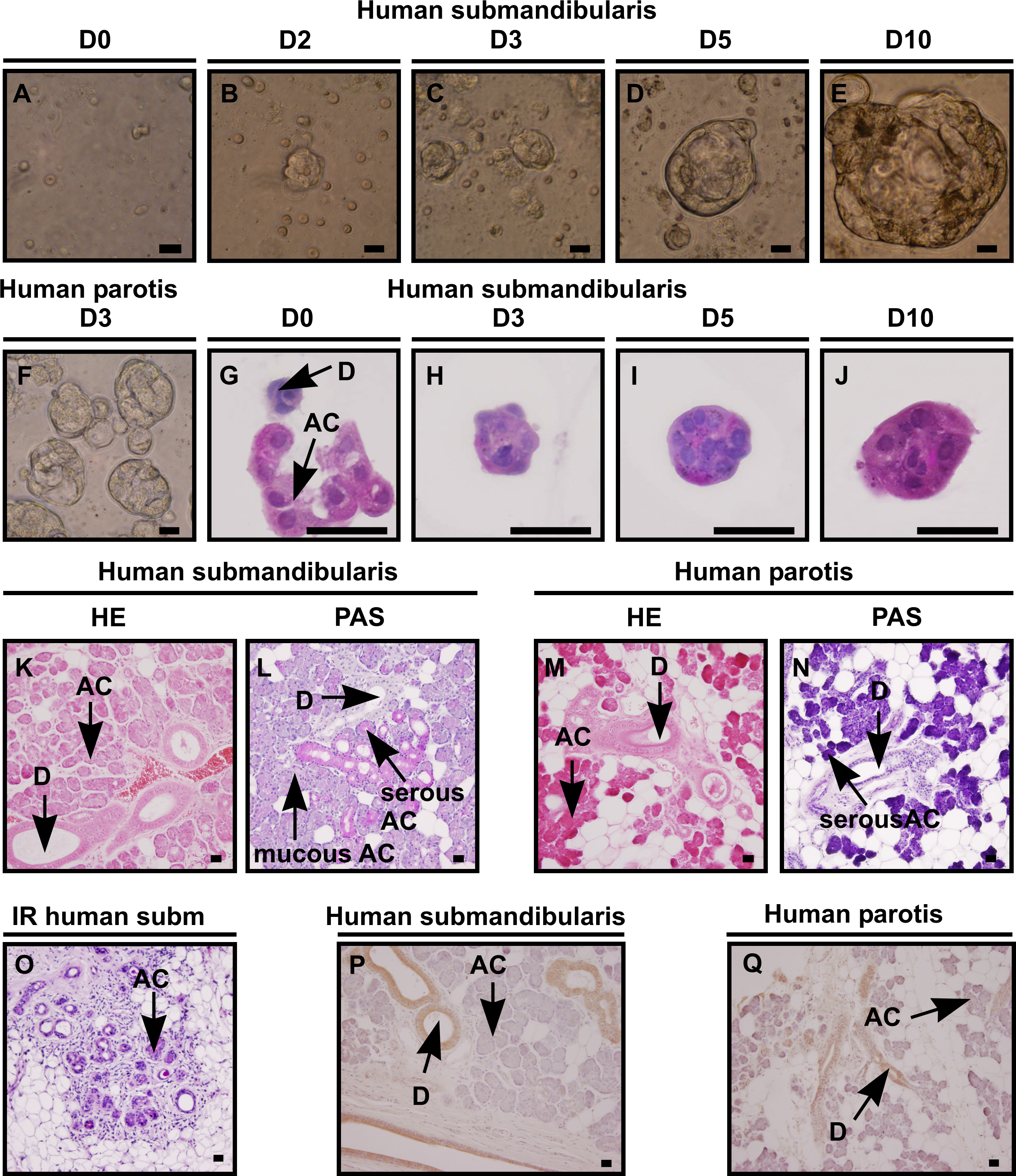

Supplement: Figure S4 — Human salivary glands contain c-kit+ cells and can yield spheres. (A–E) Dissociated human submandibular and parotid (F) glands form spheres. Initially these cells lacked mucins (PAS−) (G,H), but they differentiate in time into mucin expressing cells (I,J, PAS+). Normal human submandibular glands (K,L) contain mucous PAS+ and serous cells, whereas normal human parotid gland contain serous acini (M,N). (O) Irradiated human submandibular gland lose acinar cells very similar to the mouse. Both human submandibular (P) and parotid (Q) glands express c-Kit exclusively in duct cells. D = ductal cell type, AC = acinar cell, D0–3–5–10 represent days in culture. Scale bar = 50 µm, inset = 20 µm. (9.12 MB DOC) [file pone.0002063.s004.tif]
